# The Effect of Bolus Volume on Hyoid Kinematics in Healthy Swallowing

**DOI:** 10.1155/2014/738971

**Published:** 2014-03-23

**Authors:** Ahmed Nagy, Sonja M. Molfenter, Melanie Péladeau-Pigeon, Shauna Stokely, Catriona M. Steele

**Affiliations:** ^1^Toronto Rehabilitation Institute, University Health Network, Toronto, ON, Canada M5G 2A2; ^2^University of Fayoum, Fayoum 63514, Egypt; ^3^New York University, New York, NY 10012, USA; ^4^University of Toronto, Toronto, ON, Canada M5V 1G7; ^5^Bloorview Research Institute, Toronto, ON, Canada M4G 1R8

## Abstract

Hyoid movement in swallowing is biomechanically linked to closure of the laryngeal vestibule for airway protection and to opening of the upper esophageal sphincter. Studies suggest that the range of hyoid movement is highly variable in the healthy population. However, other aspects of hyoid movement such as velocity remain relatively unexplored. In this study, we analyze data from a sample of 20 healthy young participants (10 male) to determine whether hyoid movement distance, duration, velocity, and peak velocity vary systematically with increases in thin liquid bolus volume from 5 to 20 mL. The temporal correspondence between peak hyoid velocity and laryngeal vestibule closure was also examined. The results show that maximum hyoid position and peak velocity increase significantly for 20 mL bolus volumes compared to smaller volumes, and that the timing of peak velocity is closely linked to achieving laryngeal vestibule closure. This suggests that generating hyoid movements with increased power is a strategy for handling larger volumes.

## 1. Introduction

Hyoid movement makes an important contribution to laryngeal elevation, laryngeal vestibule closure, and upper esophageal sphincter opening during swallowing [1–4]. On a lateral view videofluoroscopic recording, the hyoid can be observed to move in a rapid burst in the superior and anterior directions, contributing to a net diagonal vector displacement [5]. These displacements are achieved by contraction of the suprahyoid floor-of-mouth muscles, while the infrahyoid strap muscles and middle pharyngeal constrictor are thought to provide stabilization and possible resistance [6]. Recent studies have confirmed that hyoid displacement distances are scaled to the size of the system [5, 7] and suggest that there is considerable variability in peak excursion measures within the healthy population [8]. Given that measures of hyoid movement distance are so variable [8–17], their utility for explaining pathophysiological mechanisms in dysphagia has come into question. However, recent studies suggest that other parameters related to hyoid movement, such as measures of hyoid movement duration [10] or displacement velocity (rate of change on position), may have clinical utility and that reduced hyoid velocities may be characteristic of swallowing impairment [11, 18, 19]. These kinematic measures can be derived from frame-by-frame hyoid position tracking across a movement trajectory [2]. Furthermore, the magnitude of peak hyoid velocity along either the vertical or horizontal planes of motion has been identified as having strong potential for classifying normal from abnormal swallows [2]. Measures of peak velocity, that is, maximum rate of position change within one plane of movement, (or* peak speed*, which is an equivalent measure of maximum rate of position change along an integrated two-dimensional vector of movement) are routinely used in studies of motor control and movement kinematics as surrogate measures of power (rate of force generation) [20–22]. It follows that measures of peak hyoid velocity may reflect the power of hyoid motion and hold functional relevance for the consequences of hyoid movement, such as laryngeal vestibule closure (LVC). To date, there is a paucity of data available describing normal hyoid velocity in swallowing and its variation according to bolus factors such as volume or viscosity. The duration of upper esophageal sphincter opening, which is biomechanically linked to hyoid movement has been shown to vary as a function of liquid bolus volume [3, 23]. It may, therefore, be reasonable to expect to see an influence of bolus volume on hyoid kinematics. Preliminary data from a recent study by Ueda and colleagues [18, 19] reported an increase in hyoid velocity but no differences in hyoid displacement measures with a doubling of liquid bolus volume. Similarly, a previous analysis from our own lab failed to identify significant changes in anatomically normalized hyoid displacement measures (i.e., distance travelled between a start and end position) between 5, 10, and 20 mL bolus volumes in healthy individuals; however, the maximum anterosuperior hyoid position was found to be further displaced relative to the C4 vertebrae for 20 mL swallows [7].

The goal of the current analysis was to characterize hyoid velocity, hyoid peak velocity, and hyoid peak speed in healthy swallowing and to determine the influence of liquid bolus volume on these parameters. The study was designed to answer the following research questions.

1. What is the impact of changing bolus volume on hyoid movement parameters (maximum displacement position, movement duration, velocity, and peak velocity), along the* X* (anterior),* Y* (superior), and* XY* (hypotenuse) axes of movement?
2. Is the timing of laryngeal vestibule closure related to any of the hyoid movement events (burst onset, end of burst, peak* X* or* Y* velocity, peak* XY* velocity, i.e., speed)?

Our hypotheses were as follows.

1. Larger bolus volumes will be associated with greater maximum hyoid positions but not with larger movement distances, as shown in a previous analysis [7].
2. Given that hyoid movement distance is not expected to vary according to bolus volume, it is expected that bolus volume will not influence hyoid movement durations or measures of hyoid velocity or peak velocity.
3. There will be a close timing correspondence between peak hyoid speed and the time of laryngeal vestibule closure.

## 2. Materials and Methods

**Figure 1:**
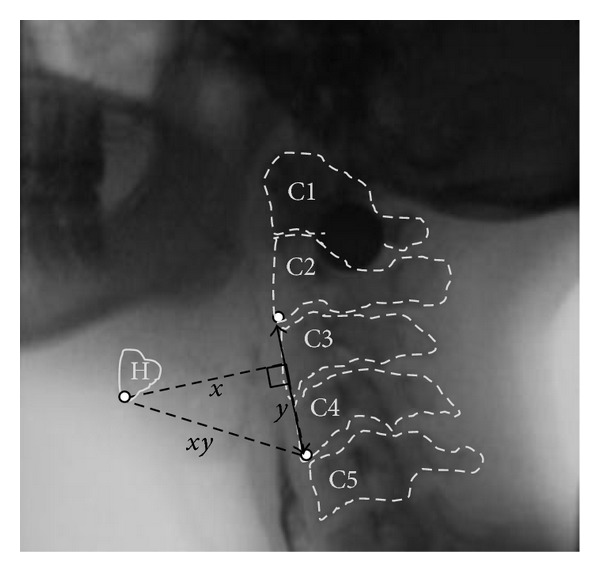
Lateral view videofluoroscopic image, showing the hyoid (*H*) at maximum excursion and the anatomical points used for measurement in a coordinate system with the origin located at the anterior-inferior corner of C4 and the vertical (*y*) axis running in a line up from the origin through the anterior-inferior corner of C2.

In this paper, we report a retrospective analysis of data from a previously reported study [23] of swallowing in 20 healthy young participants (10 male, 10 female; mean age 31.5 years, SD 5.7 years). Participants were recruited to represent the normal height distribution in the population. The data for the current analysis comprised 9 boluses of 22% w/v ultrathin liquid barium per participant, organized in blocks of 3 boluses at each of 3 target volumes (5, 10, 20 mL). Boluses were self-administered from 30 mL volume medicine cups and the order of bolus volume blocks was randomized. Precise methods for ensuring volumetric control and measuring sip volume have been described elsewhere [23]; 95% confidence intervals for actual sip volume were 3.4–3.7 mL for the 5 mL condition, 7.8–8.3 mL for the 10 mL condition, and 16.8–17.9 mL for the 20 mL condition. For simplicity, the bolus volumes will be referred to as 5, 10, and 20 mL throughout this paper. Swallows were captured in lateral view using a Toshiba Ultimax Fluoroscope (Toshiba America Medical Systems, Inc., Tustin, CA) at 30 pulses per second and recorded at 30 frames per second. Data processing involved splicing of the videofluoroscopy recordings into clips capturing a time interval beginning 30 frames prior to the first bolus of each block passing the mandibular ramus until 30 frames after the hyoid returned to rest after the 3rd bolus in the block. Data analysis involved frame-by-frame tracking of hyoid position in ImageJ freeware, using a coordinate system with an origin at the anterior-inferior corner of the C4-vertebrae and vertical axis defined by a line running from the origin upwards through the anterior-inferior corner of the C2-vertebrae (see Figure 1). The distance between the anterior-inferior corners of the C2 and C4 vertebrae also served as an anatomical scalar to enable us to control for differences in the size of the pharynx across participants during measurements of hyoid movement distance [7]. Using the frame-by-frame position histories, an algorithm in Excel VBA software was used to index the onset and end of the anterosuperior hyoid burst movement for each swallow. The onset of the hyoid burst movement was defined as the lowest position between the start of marking (10 frames before observed hyoid movement) and the peak hyoid position, calculated in both *X* and *Y* directions. The end of hyoid burst movement was defined as the peak of maximal hyoid position within the hyoid position history, calculated in both *X* and *Y* directions. This permitted the derivation of measures of maximum hyoid displacement (in anatomically normalized units, i.e., % of the C2–4 vertebral distance), distance travelled (maximum displacement minus onset position, in % C2–4 units), burst movement duration (ms), and velocity or speed (i.e., distance/duration in mm/s) for the *X*, *Y*, and *XY* movement directions. The frame (% of burst movement duration) and value (mm/s) of peak velocity or speed were also identified for each movement direction. Finally, the first frame showing laryngeal vestibule closure, defined as a seal between the laryngeal surface of the epiglottis and the arytenoids, was tracked for each swallow and its temporal location was calculated as a % of the duration of the hyoid burst movement (e.g., a value of 50% would indicate that the LVC occurred half-way through the hyoid burst movement, while measures of 30% and 70% would reflect earlier and later laryngeal vestibule closure, resp.). Strong inter- and intrarater reliability was obtained for all measurements based on repeat rating of a random selection of 10% of the recordings, as reported elsewhere [7, 23]. Statistical analyses were performed using IBM SPSS Statistics version 21. A mixed model analysis of variance (ANOVA) with a within-participant repeated factor of trial within bolus volume was performed to identify the impact of bolus volume on the study parameters. The correspondence between the timing of peak velocity and LVC, both expressed as % of the duration of the hyoid burst movement, was determined using scatter plots and correlation analysis.

## 3. Results

Table 1 summarizes descriptive statistics for the five hyoid kinematic parameters of interest by movement direction and bolus volume. Results for maximum hyoid displacement have been reported previously [7] and showed that maximum *XY* position was significantly greater for the 20 mL volume, compared to the 5 mL and 10 mL volumes *F*(2, 143.9) = 5.5, *P* = 0.005. The effect size for this comparison was small (*d* = 0.47). Maximum position captured in a single plane, that is, either the anterior (*X*) or superior (*Y*) directions of movement, did not show statistically significant variation across bolus volume.

Hyoid burst movement distance from onset to maximum displacement position did not show any significant variations for any movement direction according to bolus volume.

Hyoid burst movement durations were not significantly influenced by bolus volume.

Measures of hyoid burst velocity (i.e., movement distance/movement duration in mm/s) reflected the absence of volume specific variation seen in the component measures described above.

Peak velocity measured in mm/s was unaffected by bolus volume in the anterior and *XY* directions. However, significantly faster peak velocities were seen in the vertical direction for the 20 mL volume, compared to those seen for the 5 mL volume, *F*(2, 154.6) = 6.56, *P* = 0.002; Cohen's *d* = 0.58 (medium effect size).

**Figure 2:**
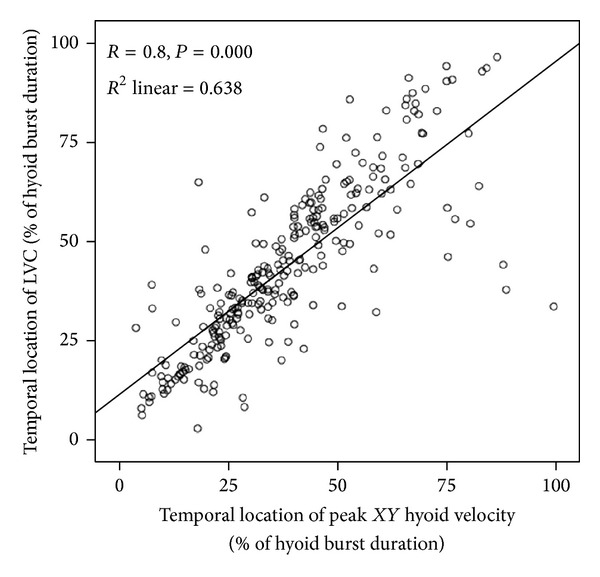
Scatter plot showing the temporal relationship between peak *xy* hyoid velocity (or speed) and the timing of laryngeal vestibule closure in thin liquid swallowing. The timing of both events is expressed relative to the timing of the hyoid burst movement. The onset of the hyoid burst movement would be represented by a value of 0% on either axis of this graph, while the end of the hyoid burst movement would be represented by values of 100% on either axis of the graph.

*Temporal Correspondence between LVC and Hyoid Peak Velocity*. The temporal location of LVC and *XY* hyoid peak velocity (i.e., hyoid peak speed) were compared using data for all bolus volumes, with both parameters expressed as a % of the hyoid burst duration. On average, the frame of LVC was located 43% into the hyoid burst movement (95% confidence interval: 41–46%), while the frame corresponding to peak *XY* velocity was located 38% into the burst movement (95% confidence interval: 36–41%). As shown in Figure 2, there was a statistically significant correlation between these events (*R* = 0.8, *P* < 0.0005, *R* ^2^ = 0.638). In other words, the earlier the peak hyoid burst velocity was achieved, the earlier the occurrence of laryngeal vestibule closure, and vice versa.

## 4. Discussion

This study provides new insights regarding hyoid movement in liquid swallowing. The data fail to show significant differences in hyoid movement distance, duration, or overall measures of velocity (distance/duration) as a function of volume, for liquids ranging from 5 to 20 mL in target volume. However, the maximum displacement position of the hyoid was further away from the anatomical origin of our measurement system for 20 mL boluses than for smaller boluses, and peak velocities were greater for the superior movement axis for these 20 mL boluses. Thus, although the distance and overall rate of change in position between hyoid starting position and maximum displacement remain stable across different volumes, larger volumes elicit movements that achieve higher peak velocities across the movement trajectory. The absence of differences in movement distance in combination with an increase in maximum displacement for the 20 mL volume implies by definition that the starting position of the hyoid at the beginning of its burst was also greater with this larger volume than for the 5 and 10 mL volumes; post hoc exploration of the data confirmed this prediction with respect to anterior starting position (*F*(2,150.16) = 6.314, *P* = 0.002) and* X*-*Y* starting position (*F*(2,150.61) = 5.18, *P* = 0.007), relative to the C4 origin of the coordinate measurement system. We interpret this finding to reflect preswallow accommodation of bolus volume in terms of hyoid positioning. In addition, the current data point to a temporal relationship between the time at which hyoid movement peak velocity occurs and the moment of laryngeal vestibule closure, and, based on this relationship, suggest that faster peak velocities will contribute to earlier LVC. If peak velocity for hyoid movement acts similarly to peak velocity for limb movement, these data suggest that larger volumes elicit hyoid movements with greater power and that associated increases in the peak velocity of hyoid burst movement may be governed by the requirement of achieving earlier laryngeal vestibule closure and airway protection. In the presence of larger volumes, it is reasonable to assume that the biological imperative to close the laryngeal vestibule might be greater, given that larger volumes may flow more rapidly into the pharynx under the influence of gravity. Future studies investigating the velocity of bolus flow as a function of volume will be needed to fully support this interpretation. Nevertheless, the close timing correspondence between peak hyoid speed and laryngeal vestibule closure suggests a functional relevance for measures of hyoid velocity.

## 5. Conclusions

Prior studies by Van Daele and colleagues and Paik et al. [2, 11] have suggested that reduced peak velocities of hyoid movement may be a characteristic of impaired swallowing. Our data provide preliminary descriptive statistics on hyoid burst kinematics for healthy swallowing of liquid boluses in the 5–20 mL range, which can serve as a reference in future studies to confirm whether disordered hyoid kinematics are seen in people with dysphagia. These data also provide preliminary support for the idea that rehabilitative techniques emphasizing higher peak velocities of submental muscle contraction for hyoid displacement may have the potential to facilitate more timely laryngeal vestibule closure. Similarly, our data point to the possibility that use of larger boluses may be an effective therapeutic method for increasing hyoid burst velocity, and thereby facilitating earlier LVC in patients who demonstrate slow closure with smaller volumes. Future studies employing methodological control of bolus volume and anatomically referenced measures of hyoid displacement and hyoid kinematics will be needed to confirm these suggestions.

## Figures and Tables

**Table 1:** 

Parameter	Direction/Axis	Bolus volume	Mean	95% confidence interval	
				Lower bound	Upper bound
Maximum hyoid displacement versus C4 origin (in % C2–4 units)	Anterior (*X*)	5 mL	133%	126%	139%
		10 mL			
		20 mL			
	Superior (*Y*)	5 mL	71%	64%	78%
		10 mL			
		20 mL			
	Hypotenuse (*XY*)	5 mL	149%	144%	155%
		10 mL			
		20 mL*	**155%**	**150%**	**161%**

Hyoid burst movement distance (in % C2–4 units)	Anterior (*X*)	5 mL	36%	33%	39%
		10 mL			
		20 mL			
	Superior (*Y*)	5 mL	43%	39%	48%
		10 mL			
		20 mL			
	Hypotenuse (*XY*)	5 mL	49%	46%	52%
		10 mL			
		20 mL			

Hyoid burst movement duration (ms)	Anterior (*X*)	5 mL	464 ms	429 ms	500 ms
		10 mL			
		20 mL			
	Superior (*Y*)	5 mL	439 ms	409 ms	468 ms
		10 mL			
		20 mL			
	Hypotenuse (*XY*)	5 mL	454 ms	418 ms	490 ms
		10 mL			
		20 mL			

Hyoid burst movement velocity (mm/s)	Anterior (*X*)	5 mL	37 mm/s	32 mm/s	41 mm/s
		10 mL			
		20 mL			
	Superior (*Y*)	5 mL	29 mm/s	25 mm/s	33 mm/s
		10 mL			
		20 mL			
	Hypotenuse (*XY*)	5 mL	49 mm/s	45 mm/s	53 mm/s
		10 mL			
		20 mL			

Hyoid burst peak velocity (mm/s)	Anterior (*X*)	5 mL	154 mm/s	141 mm/s	167 mm/s
		10 mL			
		20 mL			
	Superior (*Y*)	5 mL	89 mm/s	78 mm/s	100 mm/s
		10 mL	99 mm/s	88 mm/s	110 mm/s
		20 mL*	**113 mm/s**	**101 mm/s**	**124 mm/s**
	Hypotenuse (*XY*)	5 mL	167 mm/s	154 mm/s	179 mm/s
		10 mL			
		20 mL			

*Statistical significance at a *P*-value of < 0.05.
